# An Effective Mobile Sensor Control Method for Sparse Sensor Networks

**DOI:** 10.3390/s90100327

**Published:** 2009-01-08

**Authors:** Kriengsak Treeprapin, Akimitsu Kanzaki, Takahiro Hara, Shojiro Nishio

**Affiliations:** Dept. of Multimedia Eng., Grad. Sch. of Information Science and Technology, Osaka Univ. 1-5 Yamadaoka, Suita, Osaka 565-0871, Japan; E-Mails: kanzaki@ist.osaka-u.ac.jp; hara@ist.osaka-u.ac.jp; nishio@ist.osaka-u.ac.jp

**Keywords:** Mobile sensor, sparse area, route construction

## Abstract

In this paper, we propose an effective mobile sensor control method, named DATFM (Data Acquisition and Transmission with Fixed and Mobile node) for sparse sensor networks. DATFM uses two types of sensor nodes, *fixed node* and *mobile node*. The data acquired by nodes are accumulated on a fixed node before being transferred to the sink node. In addition, DATFM transfers the accumulated data efficiently by constructing a communication route of multiple mobile nodes between fixed nodes. We also conduct simulation experiments to evaluate the performance of DATFM.

## 1. Introduction

Recent advances in wireless communication technologies have led to an increasing interest in *ad hoc* networks that are constructed of only wireless terminals that play the role of a router. Especially, sensor networks have emerged as an important application of *ad hoc* networks. Due to the ability to construct a large-scale sensing system by cooperative behaviors of multiple sensor nodes, sensor networks are expected to be applied to many applications such as environmental monitoring, investigation of ecological system and building management.

In these areas there are some applications where it is difficult to deploy a large number of sensor nodes such as disaster sites, planetary exploration, polluted areas and underwater [1-3]. In such environments, the deployment of sensor nodes becomes too sparse to achieve sufficient sensing and data transfer. For example, in a planetary or underwater exploration, a large number (e.g. hundreds or thousands) of nodes cannot be deployed because of space and cost constrains. Moreover, in a polluted plant, the sensing area is too large to deploy a sufficient number of nodes. Although some studies assume applications where a large number of nodes are deployed from the air (e.g. from airplanes or helicopters), such a deployment becomes impossible in a building or under the heap of ruins. Furthermore, long range radio waves cannot improve the connectivity in these applications, since it is affected by the ambient surrounding such as obstacles and landscape.

On the other hand, with the development of robotics technologies in recent years, there have been many studies on sensor nodes with a moving facility (*mobile sensors*). Mobile sensors are well suited for a sparse network since a large area can be monitored with a small number of sensor nodes. In this paper, we call sensor networks which fully or partially include mobile sensors as *mobile sensor networks*.

Although many data transfer methods which utilize mobile sensors have been proposed [4-6], these methods assume an environment where the density of nodes is high. Thus, these methods cannot work well in a sparse network we assume in this paper. On the other hand, in [7-9], the authors proposed a data transfer method with mobile sensors that can transfer data even in a sparse network. However, since data transfer is performed mainly by the movement of mobile sensors, the performance of sensing and data gathering becomes significantly low especially in a sparse network.

In order to reduce the movement of mobile sensors, it is effective to construct a communication route for transmitting data to the sink node by using multiple mobile sensors. However, in a sparse network, it is difficult to construct a communication route due to the low connectivity of mobile sensors. Therefore, an effective mobile sensor control method is necessary to construct a communication route in a sparse network.

In this paper, we propose DATFM (Data Acquisition and Transmission with Fixed and Mobile node), which is an effective mobile sensor control method for sparse sensor networks to improve the efficiency of sensing and data transfer. In order to achieve effective control of mobile sensors, DATFM uses two types of sensor nodes, *fixed node* and *mobile node*. A fixed node has two roles, temporarily accumulating data acquired by nodes and constructing a communication route between fixed nodes for transmitting the accumulated data. By using the fixed nodes, DATFM can effectively control the behaviors of mobile nodes, and thus, effective sensing and data transfer can be achieved even in a sparse network.

The reminder of this paper is organized as follows. In Section 2, we briefly introduce the system model assumed in this paper and the conventional data transfer methods. In Section 3, we explain our proposed method. The results of simulation experiments are presented in Section 4. Finally, we conclude this paper in Section 5.

## 2. System model and related work

In this section, we introduce the system model assumed in this paper and the conventional studies on data transfer in sparse sensor networks.

### 2.1. System model

In this paper, we assume an application which monitors a vast area with a small number of nodes. For example, in an investigation of the ocean floor or the planetary, detection of the mineral vein or undiscovered resources in an unexplored area, and observation of the toxic gas in a disaster site, a vast area should be monitored by a small number of nodes. To monitor the whole area with a small number of nodes, mobile sensors are introduced into the network. In addition, we assume that each sensor acquires data whose sizes are relatively large such as pictures or movies.

### 2.2. Related work

Until now, many data transfer methods in mobile sensor networks have been proposed [4-12]. In [4, 6], a simple and efficient data transfer method named DFT-MSN (Delay/Fault Tolerant Mobile Sensor Network) has been proposed. In DFT-MSN, mobile sensors are classified into two types of nodes, mobile sensor nodes and high-end sink nodes. The high-end sink nodes are deployed at strategic locations where mobile sensor nodes visit with high probability. In addition, they may directly connect to the sink node all the time by changing their transmission power if necessary. Each mobile sensor node acquires data and sends it to a nearby high-end sink node by flooding with a probability in order to prevent duplication of transmitted data. After that, the high-end sink node transmits the received data to the sink node. This method assumes that each high-end sink node has to connect to the sink node all the time. As mentioned in Section 1, this assumption cannot be applied to the applications assumed in this paper.

In [5], the authors have proposed an algorithm which governs the behaviors of nodes in order to minimize the total energy consumption for moving and communication. In this algorithm, all nodes have an associated clock cycling and each node determines with a probability whether it acquires data or transmits data acquired by other nodes in every cycle. In the latter case, the node moves to construct a communication route between the sink node and a node that determines to transmit data. By doing so, this algorithm can construct the communication route to the sink node and transfer the acquired data by autonomous behaviors of nodes. However, this algorithm may not work in a sparse network because it becomes difficult to construct a communication route due to the low connectivity of nodes.

In [10], the authors assumed an environment in which mobile sensors randomly move around in an area without any control and proposed a method to determine a mobile sensor for transferring data to the sink node. In this method, each mobile sensor periodically sends information on its location to nearby sensors. Using this information, each mobile sensor predicts the locations of other sensors in the future and determines a mobile sensor to send the data. However, since mobile sensors have to periodically exchange information on their locations, this method may not work in a sparse network due to the low connectivity between mobile sensors.

In [11], the authors proposed a clustering algorithm which divides an area into several zones and clusters mobile sensors based on the divided zones. In this method, each mobile sensor periodically sends information on the frequency that it traverses the borders between zones. The mobile sensor with the smallest frequency among all sensors in a zone is elected as the cluster head. The cluster head in each cluster acts as a router in the network and transmits the data acquired in the corresponding cluster to the cluster head in an adjacent zone to which the multi-hop communication route is the most stable. This algorithm also has to periodically exchange information in order to elect the cluster heads. In addition, the data is transferred to the sink node via multi-hop communication routes between cluster heads. Thus, this algorithm may not work in a sparse network.

In [12], a data gathering method considering a sparse network has been proposed. This method utilizes a broadcasting system to control the movement of mobile sensors. Specifically, the information on the locations of mobile sensors connected with the sink node by single or multi-hop communication route is broadcasted to all mobile sensors. By using this broadcasted information, each mobile sensor can adjust the moving destination in order to decrease the moving distance to connect with the sink node or a mobile sensor which has already connected with the sink node. Hereby, data gathering from all mobile sensors can be achieved with small moving costs. However, since this method needs a broadcasting system, it is difficult to be applied to environments we assume. For example, in a building or underwater, it is generally difficult for mobile sensors to surely receive broadcasted information.

In [7, 8], RAMOS (Routing Assisted by Moving Objects) has been proposed as a data transfer method with mobile sensors. RAMOS defines several modes (classified into three categories listed below) for each sensor. Each sensor autonomously controls its behavior by changing its mode according to the existence of data.

#### Category 1: Fixed

A sensor does a sensing operation without moving. If a neighboring sensor which is within the communication range locates closer to the sink node, the sensor transmits the data to the neighboring sensor.

#### Category 2: Moving

A sensor does a sensing operation and transfers the data by moving to the sink node.

#### Category 3: Transmitting

A sensor moves around the area to find other sensors that hold data. When it connects to such a sensor, it receives and transfers the data by moving to the sink node.

In RAMOS, each sensor transfers acquired data to the sink node by changing its mode autonomously. However, since each sensor has to move to the sink node to transfer acquired data in most cases, moving cost much increases. Moreover, in a sparse network, since each sensor has few opportunities to connect to other sensors, the efficiency of sensing and data transfer becomes low.

In [9], the authors have proposed a sensing method using uncoordinated mobile nodes (UM nodes). In this method, each UM node acquires data until the amount of the acquired data reaches the memory capacity. Moreover, each UM node exchanges information on the acquired data with a connected UM node and deletes the data which were acquired by the connected node at the same location and time. By doing so, duplicated sensing (sensing a location by multiple nodes) can be suppressed and more data can be accumulated in the memory space of a UM node. In addition, this method proposes two mobility models of UM nodes, the multi-homed random way point model and the controlled mobile nodes model. In multi-homed random way point model, each UM node randomly chooses the destination and moves there. After reached the destination, it stochastically determines whether it returns to the sink node or it moves to a new destination. However, since each UM node selects the destination randomly from the whole area, the moving distance to the destination tends to increase. Thus, the efficiency of sensing decreases especially in a wide area. On the other hand, in the controlled mobile node model, the moving path of each UM node is determined in advance and each node does a sensing operation while moving its path. Furthermore, by setting moving paths of UM nodes to reduce unnecessary movement, the efficiency of sensing can be further improved. However, it is necessary to calculate the moving paths of all UM nodes in advance. Thus, the computational cost becomes very large. Moreover, this model cannot handle dynamic changes of conditions such as the existence of obstacles. Thus, moving paths cannot be determined in an unknown or highly dynamic area (e.g. planetary or underwater).

## 3. DATFM (Data Acquisition and Transmission with Fixed and Mobile node)

In this section, we explain our proposed mobile sensor control method, DATFM.

### 3.1. Assumptions

In this paper, we assume a sensor network in a wide area which is constructed by a small number of mobile sensors. The data acquired by sensor nodes are transferred to the sink node located at a corner of the area similar to the assumption in many conventional works such as target detecting, tracking and environment monitoring [9, 13-14]. Each node has a unique identifier in the network. In addition, we assume that all nodes have the same sensor and radio devices. Thus, the sensing and wireless communication ranges are same among all nodes.

Following the conventional works [4-12], we assume that there are no obstacles in the area. There are two types of sensor nodes, *fixed node* and *mobile node* (the sink node is classified as a fixed node). Each node acquires its present location by using GPS or other location detection methods [14-16]. Moreover, all nodes know the locations of all fixed nodes. This assumption is valid in applications where the locations of fixed nodes are strategically decided before deploying them.

#### 3.1.1. Fixed node

A fixed node does not move. It has larger memory capacity, compared with a mobile node, and accumulates data acquired by itself and from other nodes. In addition, it controls nearby mobile nodes to construct a communication route when transmitting the accumulated data toward the sink node. Furthermore, it holds information on nearby mobile nodes that directly connected to it (entered the wireless communication range) for a certain period; the information includes the identifier of each mobile node, the time when the node connected to it, the fixed node that the node goes to next, and the next destination (sensing point or location of the next fixed node to go) of the node. Table 1 shows an example of the information held by a fixed node. Each fixed node sets the validity period for each record in the information (each row in Table 1), and removes it when the validity period expires. Here, the validity period for mobile node *i* is calculated by the following equation:

(1) $${\text{VP}}_{\text{i}}=\frac{\sqrt{{\text{S}}_{\text{area}}}}{\upsilon_{\text{m}}}.$$

In the above equation, ***S****_area_* and ***υ****_m_* respectively denote the area size and the velocity of mobile nodes in the sensing mode (described in the next subsection). By using this information, each fixed node predicts where each mobile node exists.

#### 3.1.2. Mobile node

A mobile node moves around the area. In addition, it has the following three modes:

##### Sensing mode (*SM*)

A node sets a destination randomly within the area and moves there. After reaching the destination, it does a sensing operation and decides its new destination.

##### Collecting mode (*CM*)

When a node in *SM* receives a *route request packet* (*RReq*) from a fixed node, it changes its mode into collecting mode (*CM*). In *CM*, a node moves faster than that in *SM* in order to collect other mobile nodes to construct a communication route.

##### Transmission mode (*TM*)

When a node in *SM* receives a *route construction request packet* (*RCReq*) from a fixed node or a mobile node in *CM*, it changes its mode into transmission mode (*TM*). In *TM*, a node constructs a route and transfers the data.

**Figure 1.**
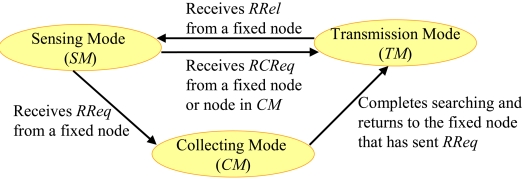
Mode transition of a mobile node.

Figure 1 shows the mode transition of a mobile node.

### 3.2. Moving strategy of mobile nodes

**Figure 2.**
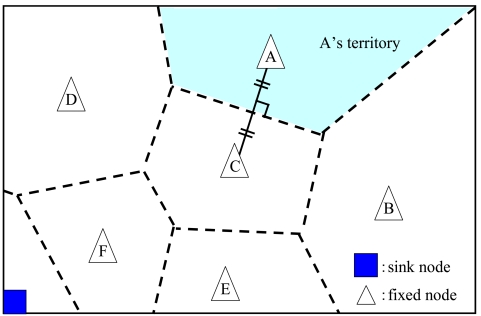
Dividing the area.

DATFM divides the area into several regions based on a Voronoi diagram in which fixed nodes are the site points. Here, the Voronoi diagram of a set of point partitions the area into convex polygons that consist of the vertical bisectors of the points. Every point in a polygon is closer to the site point in the corresponding polygon than to any other site points. In DATFM, each site point (a fixed node) has charge of the corresponding region. In other words, each fixed node has a role for collecting data acquired in the region the fixed node exists. We call the region for each fixed node as its *territory*. Figure 2 shows an example of Voronoi diagram and divided territories

**Figure 3.**
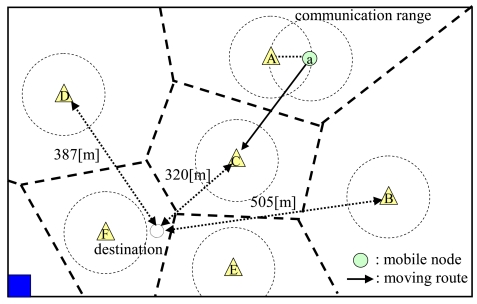
Moving route of a mobile node.

A mobile node basically sets its mode as *SM* and moves to the selected destination by the following steps:

1. It moves to connect to the nearest fixed node (i.e. the fixed node in the current territory) and transmits its acquired data.
2. It calculates the distances between its destination and all fixed nodes in the adjacent territories and moves to the fixed node that is the nearest to its destination. In addition, it sends information on its identifier, its next fixed node to move, and its destination to the connecting fixed node before moving to the next fixed node. Figure 3 shows an example to choose the next fixed node to move. After transmitting its acquired data to fixed node ***A***, the mobile node ***a*** calculates the distances between its destination and fixed nodes ***B, C***, and ***D***, which are in territories adjacent to ***A***'s territory. After that, it chooses fixed node ***C*** that is the nearest to the destination and moves there. These procedures are repeated until the mobile node connects to the fixed node that has charge of the territory which contains the destination.
3. It moves to its destination and does a sensing operation.

This moving strategy enables each fixed node to have many opportunities to connect to mobile nodes. Moreover, each fixed node can predict the locations of the recently connected mobile nodes by using the information shown in Table 1. Therefore, this strategy makes it easy for fixed nodes to collect mobile nodes in the data transmission phase described in the next subsection.

### 3.3. Data Transmission

A fixed node starts to transmit the accumulated data when the amount of the accumulated data in its memory exceeds the predetermined threshold. In what follows, we explain the procedures for transferring the accumulated data to the sink node.

#### 3.3.1. Selection of the next fixed node

The fixed node that starts data transmission (the source node) selects a next fixed node to transmit the data (the destination node) by using the Delaunay triangulation. The Delaunay triangulation can be performed by connecting the site points in the Voronoi diagram whose polygons share a common edge.

**Figure 4.**
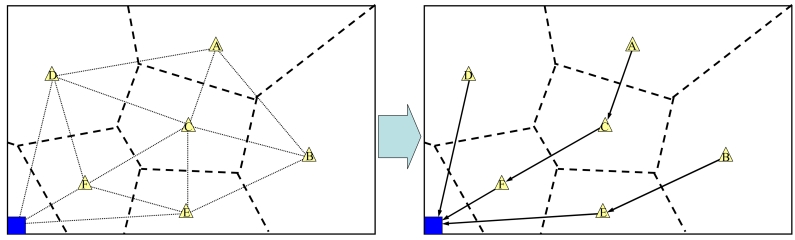
Selection of the next fixed node.

The source node creates Delaunay triangles which include itself, and selects another fixed node that is a vertex of a Delaunay triangle and is the nearest to the sink node. This node is set as the destination node. When the sink node is a vertex of the created Delaunay triangles, the source node selects the sink node as the destination. Figure 4 shows an example of selecting the destination node. Fixed node ***A*** selects the destination node from fixed nodes ***B, C*** and ***D***. In this figure, since fixed node ***C*** is the nearest to the sink node, it becomes the destination node. On the other hand, when fixed node ***D*** starts data transmission, it selects the sink node as the destination node since the sink node is a vertex of the created Delaunay triangles.

#### 3.3.2. Request for collecting mobile nodes

The source node firstly checks whether a communication route to the destination node exists. If it does, the source node transmits the data to the destination node via the communication route. Otherwise, the source node sends *RReq* to a mobile node that firstly connects to it. If multiple mobile nodes already connect to the source node, the source node randomly selects one of them and sends *RReq* to it. The mobile node that receives the *RReq* changes its mode into *CM*. Here, a *RReq* includes the identifiers of the source and destination nodes, the *required number of mobile nodes* ***N****_req_*, and the *time limit* ***T***_lim_ for collecting mobile nodes. ***N****_req_* is the number of mobile nodes which is required to construct the communication route, and is calculated by the following equation:

(2) $${\text{N}}_{\text{req}}=\left\lfloor \frac{\left|L_{\text{src}}-L_{\text{dst}}\right|}{{\text{R}}_{\text{com}}}\right\rfloor .$$

**Figure 5.**
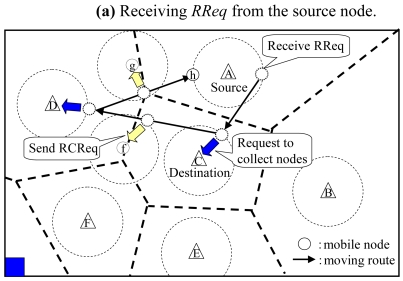

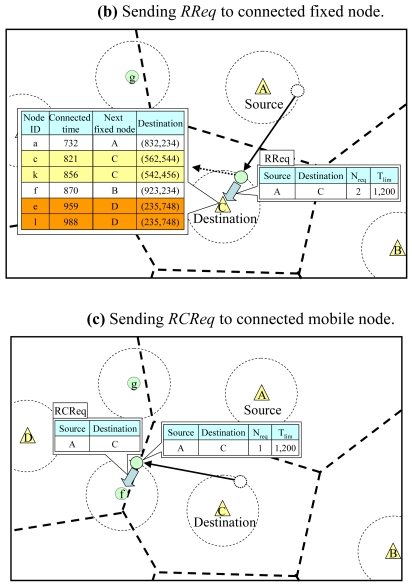


***L****_src_* and ***L****_dst_* are the locations of the source and destination nodes. ***R****_com_* is the wireless communication range. ***T***_lim_ is the time that terminates collecting mobile nodes. The detail of the time limit is described in Subsection 3.3.4. Figure 5(a) shows an example of *RReq* sent from the source node ***A***.

The mobile node in *CM* travels around the nearby fixed nodes to collect the other mobile nodes. First, the mobile node in *CM* moves to the destination node. When the mobile node connects with a fixed node, it does the following procedures:

1. It calculates the number of mobile nodes which are performing a sensing operation in the territory of the connected fixed node. This can be done by checking the ‘next destination’ record in the information held by the fixed node. Specifically, when the ‘next destination’ is in the territory of the fixed node, the corresponding mobile node is performing the sensing operation in the territory.
2. It decreases ***N****_req_* in the *RReq* by that calculated number of nodes. This is because the mobile nodes performing a sensing operation in a territory will connect to the fixed node in the territory after the sensing operation. As described later, those mobile nodes will receive *RCReq* from the fixed node, and move to the source node to help data transmission.
3. It sends the updated *RReq* to the fixed node.
4. It chooses the next fixed node to go. Specifically, it checks the ‘next fixed node’ record in the information held by the fixed node, and chooses one to which the largest number of mobile nodes have gone, and which it has not visited yet.

Here, the mobile node in *CM* goes back to the source node when at least one of the following conditions is satisfied:

- The next fixed node to go cannot be found.
- ***N****_req_* in *RReq* becomes zero.
- The time limit has come.

Figure 5(b) shows an example of behaviors of a mobile node in *CM* connected to fixed node ***C***. It calculates the number of mobile nodes which are performing a sensing operation in the territory of fixed node ***C***. In this figure, the mobile node in *CM* knows that mobile nodes ***c*** and ***k*** are performing a sensing operation since their next fixed node is ***C***. Thus, the mobile node in *CM* decreases ***N****_req_* in the *RReq* by two and sends the updated *RReq* to fixed node ***C***. After that, it checks the ‘next fixed node’ record in the information held by fixed node ***C***, and chooses fixed node ***D*** as the next destination to go since the largest number of nearby mobile nodes (i.e., ***e*** and ***l***) have gone.

After returning to the source node, the mobile node in *CM* changes its mode. If the required number of mobile nodes has not been collected, the node in *CM* changes its mode into *TM*. Otherwise, if ***N****_req_* mobile nodes have been collected and the communication route have been constructed, it changes back to *SM* and returns to a sensing operation.

When the mobile node in *CM* connects to other mobile nodes while moving, it sends a *RCReq* to the connected nodes. Here, a *RCReq* includes the identifiers of the source and destination nodes. When the mobile node that receives the *RCReq* is in *SM*, it moves to the source node and changes its mode into *TM*. After that, the mobile node in *CM* decreases the ***N****_req_* in the *RReq* by one. For example in Figure 5(c), when a mobile node in *CM* connects with mobile node ***f***, it sends a *RCReq* to ***f*** and decreases ***N****_req_*. On the other hand, when a mobile node in *SM* connects to the source node or a fixed node which received the *RReq* from a mobile node in *CM*, the source node or the fixed node sends a *RCReq* to the connected mobile node. The mobile node that receives the *RCReq* changes its mode into *TM* and moves to the source node. Here, a fixed node which received the *RReq* from the mobile node in *CM* continues to send *RCReq*s until the time limit comes.

#### 3.3.3 Data transmission using the collected node

The source node starts data transmission when it firstly connects to a mobile node in *TM*. First, the source node calculates the location, (***x****_k_*, ***y****_k_*), of each collected mobile node ***m****_k_* by the following equations:

(3) $${\text{x}}_{\text{k}}={\text{x}}_{\text{src}}+\frac{\text{k}}{\text{n}+1}\cdot ({\text{x}}_{\text{dst}}-{\text{x}}_{\text{src}}).$$

(4) $${\text{y}}_{\text{k}}={\text{y}}_{\text{src}}+\frac{\text{k}}{\text{n}+1}\cdot ({\text{y}}_{\text{dst}}-{\text{y}}_{\text{src}}).$$

**Figure 6.**
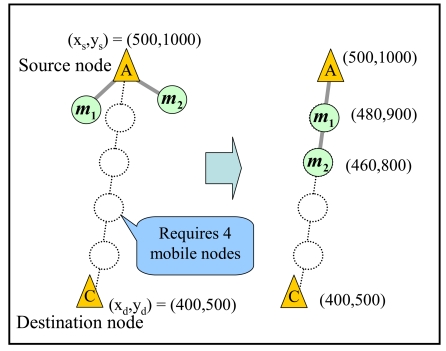
Construction of a train.

Here, ***n*** is the number of collected mobile nodes and each of the collected mobile nodes is assigned an identifier (***m*_1_, *m*_2_, …, *m****_n_*). (***x****_k_*, ***y****_k_*) and (***x****_dst_*, ***y****_dst_*) are the locations of the source and destination nodes. Then, the source node sends information on the calculated locations to the collected mobile nodes. The collected mobile nodes move to their locations. Figure 6 shows an example of calculating the locations of mobile nodes when two mobile nodes are collected.

Here, if the sufficient number of mobile nodes are collected, the communication route between the source and destination nodes is constructed. Otherwise, if the number of collected mobile nodes is smaller than the required number ***N****_req_*, the source node transmits the data by using *train transmission*.

In train transmission, data are transferred by cooperative movement and communication among the collected mobile nodes. Specifically, the collected mobile nodes firstly form a line segment by the above mentioned process. After that, the collected nodes repeat the following procedures:

1. The source node transmits a part of the accumulated data so that the amount of the transmitted data equals to the sum of memory spaces of the collected nodes.
2. The collected nodes move toward the destination node with keeping same distances between adjacent nodes, and stop when the node at the other end of the line segment connects to the destination node.
3. The collected nodes transmit all the data to the destination node through the line segment (communication route), and then, move back toward the source node.

**Figure 7.**
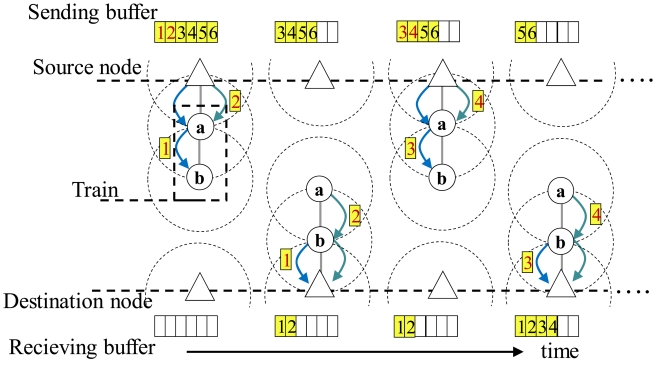
Train transmission.

Figure 7 shows an example of train transmission with two mobile nodes.

**Figure 8.**
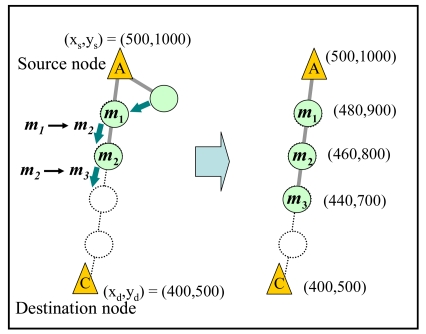
Reconstruction of a train.

Moreover, when another mobile node in *TM* connects to the source node after started the train transmission, the source node adds the connected mobile node to the ‘train’. Specifically, the source node increments the identifiers of mobile nodes (i.e. ***m****_k_* **→ *m****_k_***_+1_**) in the current train and assigns identifier ***m*_1_** to the newly connected node. Then, the source node recalculates the location of each mobile node and sends information on the recalculated locations to all the collected mobile nodes. The mobile nodes which received the information move to their new locations and restart the train transmission. Figure 8 shows an example when another mobile node connects the source node. In this figure, the newly connected node is assigned identifier ***m*_1_** and added to the train.

Here, when the number of the collected nodes reaches ***N****_req_*, the completed communication route is constructed. In that case, the source node stops the train transmission and start transmitting the data via the constructed route. Moreover, the source node sends a *route release packet* (*RRel*) to nodes in *TM* which newly connects after constructed the complete communication route. The mobile nodes that received the *RRel* change their mode into *SM* and restart moving to their destinations.

**Figure 9.**
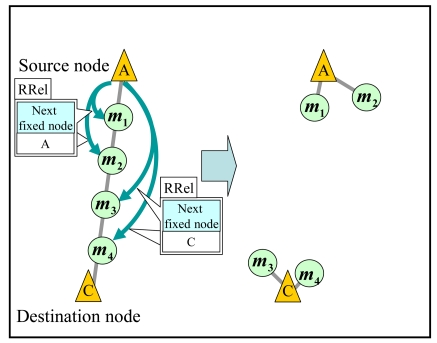
Release a communication route.

After transmitting all the accumulated data, the source node sends a *RRel* to each mobile node in *TM*. A *RRel* includes the information on the next fixed node to go. Each mobile node that receives the *RRel* changes its mode into *SM* and moves to the destination specified in the *RRel*. Here, the source node sets the next fixed node for the half of mobile nodes in *TM* which are far from itself as the destination node of the data transmission. This helps the destination node to collect mobile nodes for the next data transmission since the amount of data in its memory space may exceed the threshold. On the other hand, for each of the other mobile nodes, the source node sets the next fixed node as itself. Figure 9 shows an example when the source node ***A*** has transmitted all the accumulated data to the destination node ***C***. The source node ***A*** sends *RRel*s including ***C*** as the next fixed node to mobile nodes ***m*_3_** and ***m*_4_**. It also sends *RRel*s including itself as the next fixed node to mobile nodes ***m*_1_** and ***m*_2_**. After that, mobile nodes ***m*_1_** and ***m*_2_** restart to move to the destination after connected to fixed node ***A***.

#### 3.3.4. Decision of the time limit

In DATFM, the source node sets the time limit and notifies it to the mobile node in *CM* and other nearby fixed nodes. As described in Subsection 3.3.2, this value is used for terminating collecting mobile nodes for the corresponding data transmission. Here, in order to set the appropriate time limit, we use the estimated time elapsed to collect the required number of mobile nodes.

In order to calculate the time limit, we assume that the following parameters are given:

**L***_i_*: the location of fixed node ***i***.
**T***_i_*: the territory of fixed node ***i***.
**d***_i_*: the location of the destination (sensing point) of a mobile node in T*_i_* (i.e. **d***_i_* ∈ **T***_i_*).
***S****_area_*: the size of the whole area (i.e. ${\text{S}}_{\text{area}}=\underset{\text{i}}{\text{∑}}\left|T_{\text{i}}\right|$).
***υ****_m_*: the velocity of mobile nodes in *SM*.
***T****_acq_*: the time for a sensing operation at a destination (sensing point).
***S****_F_*: the set of fixed nodes in the whole area.
***N****_mov_*: the number of mobile nodes in the whole area.
***N****_reqi_*: the required number of mobile nodes to construct the completed communication route from fixed node ***i***.

Let us denote ***T****_esti_* as the estimated time in which the required number of mobile nodes connect to fixed node ***i***. Then, the time limit of fixed node ***i*** (***T*_lim_***_i_*) is calculated by the following equation:

(5) $${\text{T}}_{{\text{lim}}_{\text{i}}}={\text{T}}_{\text{current}}+{\text{T}}_{{\text{est}}_{\text{i}}}.$$

Here, ***T****_current_* is the current time. In what follows, we describe how to calculate ***T****_esti_*.

First, let us denote ***T****_avei_* as the average interval for fixed node ***i*** that mobile nodes connect to ***i***. By using this value, we can derive the following equation:

(6) $${\text{T}}_{{\text{est}}_{\text{i}}}={\text{N}}_{\text{req}}\cdot {\text{T}}_{{\text{ave}}_{\text{i}}}.$$

In order to calculate ***T****_avei_*, we define *sensing cycle* of a mobile node engaged in a sensing operation in ***T****_i_*. The *sensing cycle* is defined as the sequence of a sensing operation in which the mobile node departs from the fixed node in the region including the previous sensing point, moves to the sensing point, performs the sensing operation, and moves to fixed node ***i***. We also define the *average sensing cycle time* ***T****_cycle_* as the average time elapsed for a sensing cycle of a mobile node that performs a sensing operation. Here, since the moving path to the fixed node which has charge of the destination (described in Subsection 3.2) can be roughly approximated as the straight line, we assume that mobile nodes go straight to the fixed node (do not go through fixed nodes in the neighboring territories) in order to simplify the calculation. Actually, the effect of the difference of moving to the analysis is small.

**Figure 10.**
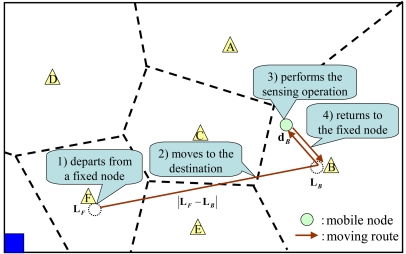
The operations in a sensing cycle.

As an example, we assume a mobile node in Figure 10 which departs from fixed node ***F*** and performs a sensing operation in ***T****_B_*. First, since the distance between fixed nodes ***F*** and ***B*** is |**L***_F_* − **L***_B_*|, the time elapsed for moving to fixed node ***B*** (***T****_movB_*) becomes |**L***_F_* − **L***_B_*|/***υ****_m_*. Next, the time elapsed for moving from fixed node ***B*** to the destination (sensing point) is |**L***_B_* − **d***_B_*|/***υ****_m_*. Finally, after the sensing operation at a destination (elapsed time is ***T****_acq_*), the time elapsed for moving back to fixed node ***B*** becomes |**d***_B_* − **L***_B_*|/***υ****_m_*. Thus, the total time elapsed of the sensing cycle becomes:

(7) $$\frac{\left|L_{\text{F}}-L_{\text{B}}\right|}{\upsilon_{\text{m}}}+\frac{\left|L_{\text{B}}-d_{\text{B}}\right|}{\upsilon_{\text{m}}}+{\text{T}}_{\text{acq}}+\frac{\left|d_{\text{B}}-L_{\text{B}}\right|}{\upsilon_{\text{m}}}=\frac{\left|L_{\text{F}}-L_{\text{B}}\right|}{\upsilon_{\text{m}}}+{\text{T}}_{\text{acq}}+2\cdot \frac{\left|d_{\text{B}}-L_{\text{B}}\right|}{\upsilon_{\text{m}}}$$

Here, since each mobile node randomly chooses the destination in the whole area, the average elapsed time for moving between fixed node ***B*** and the destination **d***_B_* becomes the average of the moving time form **L***_B_* to any location in **T***_B_*. Due to the same reason, the probability that a mobile node departs from fixed node ***F*** depends on the ratio of the size of **T***_F_* (|**T***_F_****|***) to the size of the whole area (***S****_area_*). Thus, the average time which a mobile node departs from any fixed node (other than ***B***) and moves to fixed node ***B*** becomes:

(8) $${\text{T}}_{{\text{mov}}_{\text{B}}}=\sum_{\text{j}\in {\text{S}}_{\text{F}},\text{j}\neq \text{B}}\frac{\left|T_{\text{j}}\right|}{S_{\text{area}}}\cdot \left(\frac{\left|L_{\text{j}}-L_{\text{B}}\right|}{\upsilon_{\text{m}}}\right).$$

Therefore, the *average sensing cycle time* in **T***_i_* (***T****_cyclei_*) is derived by the following equation:

(9) $${\text{T}}_{{\text{cycle}}_{\text{i}}}=\sum_{\text{j}\in {\text{S}}_{\text{F}},\text{j}\neq \text{i}}\frac{\left|{\text{T}}_{\text{j}}\right|}{S_{\text{area}}}\cdot \left(\frac{\left|L_{\text{i}}-L_{\text{j}}\right|}{\upsilon_{\text{m}}}\right)+T_{\text{acq}}+2\cdot \frac{\text{ave}\left(\left|d_{\text{i}}-L_{\text{i}}\right|\right)}{\upsilon_{\text{m}}}.$$

Since a mobile node chooses its destination randomly from the whole area, the probability that a mobile node chooses the destination in **T***_i_* becomes the ratio of the size of **T***_i_* (|**T***_i_*|) to the size of the whole area (***S****_area_*). Thus, the average sensing cycle time in the whole area (***T****_cycle_*) is derived by the average of the ***T****_cyclei_* for all ***i***s.

(10) $${\text{T}}_{\text{cycle}}=\sum_{\text{j}\in {\text{S}}_{\text{F}}}\frac{\left|{\text{T}}_{\text{i}}\right|}{S_{\text{area}}}\cdot T_{{\text{cycle}}_{\text{i}}}.$$

Since the mobile node connects to the fixed node after the sensing operation, all mobile nodes connect to a fixed node in the period of ***T****_cycle_*. Furthermore, since the number of mobile nodes which choose the destination in **T***_i_* is ***N****_mov_* · |**T***_i_*|/***S****_area_*, we can estimate that ***N****_mov_* · |**T***_i_*|/***S****_area_* mobile nodes connect to fixed node ***i*** in the period of ***T****_cycle_*. Thus, the average time in which a mobile node connects to fixed node ***i*** (***T****_avei_*) is calculated by the following equation:

(11) $$\begin{array}{ll} {\text{T}}_{{\text{ave}}_{\text{i}}} & =\frac{{\text{T}}_{\text{cycle}}}{\frac{{\text{N}}_{\text{mov}}\cdot \left|{\text{T}}_{\text{i}}\right|}{{\text{S}}_{\text{area}}}}\\ & =\frac{T_{\text{cycle}}\cdot {\text{S}}_{\text{area}}}{{\text{N}}_{\text{mov}}\cdot \left|T_{\text{i}}\right|}. \end{array}$$

Based on the above discussion, the time limit (***T*_lim_***_i_*) can be calculated by the following equation:

(12) $$\begin{array}{ll} {\text{T}}_{{\text{lim}}_{\text{i}}} & ={\text{T}}_{\text{current}}+{\text{T}}_{est_{\text{i}}}\\ & ={\text{T}}_{\text{current}}+{\text{N}}_{{\text{req}}_{\text{i}}}\cdot {\text{T}}_{{\text{ave}}_{\text{i}}}\\ & =T_{\mathrm{current}}+{\text{N}}_{{\text{req}}_{\text{i}}}\cdot \frac{T_{\text{cycle}}\cdot {\text{S}}_{\text{area}}}{{\text{N}}_{\text{mov}}\cdot \left|T_{i}\right|}. \end{array}$$

### 3.4. Discussions

DATFM can acquire and transfer data efficiently due to the following features:

- The source node can collect mobile nodes easily since each mobile node travels around the fixed nodes as described in Subsection 3.2.
- Since each mobile node transmits the acquired data to the nearest fixed node, it can store new data more frequently.
- Since the accumulated data are transmitted by using multiple mobile nodes, the data can reach to the sink node easily even in a wide area.
- By applying train transmission, the source node can transmit the accumulated data even when the distance between the source and destination nodes is long.

Here, as described in Subsection 3.1, DATFM assumes a flat environment where no obstacles exist. However, even when obstacles exist in the area, DATFM can work by letting each fixed node hold the information on obstacles in its territory and adjacent territories. By doing so, the source node of a data transmission process can determine an appropriate communication route considering the obstacles.

In DATFM, fixed nodes have larger memory spaces than mobile nodes. If mobile nodes have same memory spaces as those of fixed nodes, fixed nodes have to start a data transmission process every time they receive data from a single mobile node. In this case, mobile nodes have to move to transfer the data towards the sink node every time they send the acquired data to a fixed node. This may cause the deterioration of the performance of sensing in DATFM. Therefore, we set the size of memory spaces of mobile nodes smaller than those of fixed nodes in order to balance the efficiencies of data transfer and sensing.

As described in Subsection 3.3.3, the source node sends a *RReq* with ***N****_req_*, which is the number of mobile nodes required to construct a communication route. Here, when a mobile node is performing a sensing operation in the territory of the source node, it will certainly connect to the source node. Also, the source node knows such nodes since all mobile nodes in the territory certainly connect to the fixed node which has charge of the destination before starting a sensing operation. Thus, the source node can decrease ***N****_req_* considering mobile nodes which are performing sensing operation in its territory. However, DATFM does not decrease ***N****_req_* in order to certainly construct a communication route even in a case that node failures occur.

In addition, the source node sends a *RReq* only to the mobile node which firstly connects after starting the data transmission process. Then, the source node starts a train transmission when the second mobile node connects. Here, if the source node sends *RReq*s to multiple mobile nodes it might be able to collect ***N****_req_* mobile nodes in a shorter time. However, the source node does not do so because it becomes difficult to correctly manage ***N****_req_*. For example, when another mobile node (node ***b***) connects to the source node after the source node has sent a *RReq* to a mobile node (node ***a***), the source node cannot set the appropriate ***N****_req_* for node ***b*** since it cannot know the number of mobile nodes collected by node ***a***. Moreover, the main task of a mobile node in *CM* is to request the nearly fixed nodes to collect other mobile nodes. This is because a single mobile node has a little chance to connect to another mobile node in a sparse network. Even if the source node sends *RReq*s to multiple mobile nodes, the effect will be little. Therefore, DATFM starts the train transmission when the second mobile node connects to the source node in order to improve the efficiency of data transmission.

## 4. Performance evaluation

In this section, we show results of simulation experiments regarding performance evaluation of DATFM. In the simulation experiments, we compare the performances of DATFM, UM with random way point model [9], and RAMOS [7,8].

### 4.1. Simulation environment

In the simulation experiments, we assume a vast agricultural farm in which each sensor acquires pictures or movies of the crops. Sensor nodes are deployed in a 1,000[m]×1,000[m] flatland. Each sensor node performs a sensing operation with the rate of 100 [***bit/sec*·*m*^2^**] and ***T****_acq_* is 30 [sec]. The wireless communication range of all nodes and the channel bandwidth are ***R****_com_* [m] and 2 [Mbps], respectively.

In DATFM, there are 10 fixed nodes and 40 mobile nodes. Each mobile node moves with velocity of ***υ****_m_* [m/s] in *SM* and **2*υ****_m_* [m/s] in *TM* and *CM*. Each fixed and mobile node has a memory space whose size is 2,000 [Mbit] and 10 [Mbit], respectively. Each fixed node starts data transmission when the amount of the accumulated data exceeds 800 [Mbit]. Each mobile node performs a sensing operation every time it arrives the destination. Each fixed node performs a sensing operation every 1,500 [sec]. The rate and the duration of a sensing operation by a fixed node are also 100 [***bit/sec*·*m*^2^**] and 30 [sec].

In RAMOS, there are 10 nodes in category1 (fixed), (40 - 10) nodes in category2 (moving), and 10 nodes in category3 (transmitting). This parameter setting is to make the total number of nodes in category2 and category3 in RAMOS equal to 40 and to guarantee all nodes in category1 can transfer data to the sink node by using nodes in category3. Nodes in category1 have a memory space of 1,000 [Mbit]. Nodes in category2 and category3 have a memory space of 10 [Mbit]. Nodes in category2 and category3 move with the velocity of ***υ****_m_* [m/s] when sensing and gathering data, and **2*υ****_m_* [m/s] when transmitting data to the sink node. The nodes in category1 perform a sensing operation every 1,500 [sec] in the same way as fixed nodes in DATFM.

In UM, there are (40 + 10) UM nodes. Each UM node has a memory space of 10 [Mbit]. Each node moves with velocity of ***υ****_m_* [m/s] when sensing, and **2*υ****_m_* [m/s] when transferring data. Each UM node starts transferring data to the sink node when the amount of the accumulated data exceeds 10 [Mbit] (i.e. each node transfers data after a sensing operation).

We set parameters ***R****_com_* and ***υ****_m_* as shown in Table 2. In the experiments, we change one of the parameters and set others as default values in order to verify the effect of each parameter. In this environment, we evaluate the following four criteria during 60,000 [sec]:

- Throughput
  The amount of data that arrive at the sink node per 1[sec].
- Average moving distance
  The average of moving distances of all mobile nodes (i.e. all mobile nodes in DATFM, all nodes except those in category1 in RAMOS, and all nodes in UM) during the simulation period.
- Average delay
  The average of the elapsed time after data are acquired until the data arrive at the sink node.
- Control traffic
  The number of packets to control sensor nodes during the simulation period. Specifically, in DATFM, the total number of *RReq*s, *RCReq*s, and *RRel*s sent by nodes becomes the control traffic. On the other hand, the control traffic in RAMOS is the total number of packets sent from nodes in

### 4.2. Effects of the velocity of node

**Figure 11.**
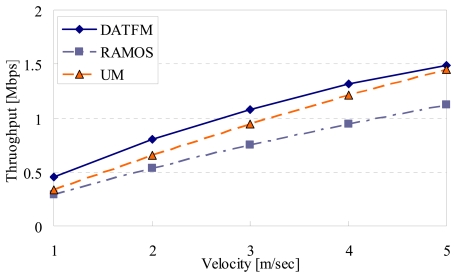
Velocity and throughput.

**Figure 12.**
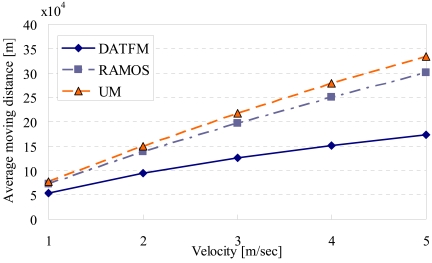
Velocity and average moving distance.

**Figure 13.**
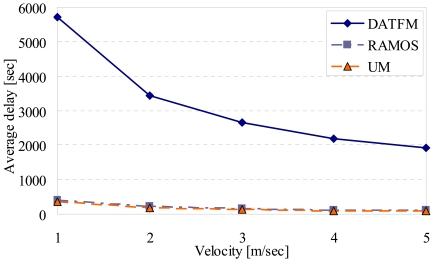
Velocity and average delay.

Figures 11, 12, 13 and 15 show the simulation results changing parameter ***υ****_m_*. The horizontal axis on all graphs indicates the velocity of sensor nodes ***υ****_m_*. The vertical axis indicates throughput in Figure 11, average moving distance in Figure 12, average delay in Figure 13, and control traffic in Figure 15.

#### 4.2.1. Throughput

Figure 11 shows that the throughput in DATFM is always larger than those in RAMOS and UM. This is because DATFM can acquire and transfer data more efficiently than RAMOS and UM. As ***υ****_m_* increases, the difference in throughput between DATFM and RAMOS gets larger. Mobile nodes in DATFM transfer the acquired data to a nearby fixed node while mobile nodes in RAMOS transfer data to the sink node every time they acquired data. Therefore, mobile nodes in DATFM can return to perform sensing operation more quickly than those in RAMOS especially when the mobile nodes move with higher speed.

On the other hand, the difference in throughput between DATFM and UM gets smaller as ***υ****_m_* increases. This is because the number of mobile nodes in DATFM (40) is smaller than that in UM (40 + 10). Thus, UM is more affected by the velocity of nodes than DATFM.

#### 4.2.2. Average moving distance

Figure 12 shows that the moving distances in all methods increase as ***υ****_m_* gets larger, which is an obvious result. In addition, the moving distance in DATFM is smaller than those in RAMOS and UM. This is because sensor nodes in RAMOS and UM move to the sink node every time they acquire data. On the other hand, since DATFM accumulates the acquired data on a fixed node before transmitting them to the sink node, the mobile node that acquires the data does not need to move to the sink node.

Furthermore, the difference in the moving distance between three methods increases as ***υ****_m_* gets larger. This is because the mobile nodes in all methods can perform much sensing operations as ***υ****_m_* gets larger, and thus, the numbers of movements between the sensing point of each sensor nodes and the sink node in RAMOS and UM become larger.

#### 4.2.3. Average delay

Figure 13 shows that the delay for transmitting data in DATFM is always larger than those in RAMOS and UM. This is because DATFM needs much time for accumulating data on fixed nodes. On the other hand, the average delays in the other methods do not change even when the number of mobile nodes increases. This is because the mobile nodes in these methods transfer data without accumulating data. As ***υ****_m_* increases, average delays of all methods decrease. This is obvious since each sensor node can transfer data faster.

**Figure 14.**
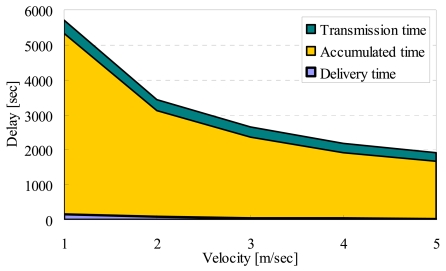
Velocity and details of delay in DATFM.

Figure 14 shows the details of delay in DATFM. In this figure, the delivery time is the time period after a mobile node acquires data until it connects to a fixed node. The accumulated time is the time period during which the data are stored at a fixed node. The transmission time is the time elapsed for transmitting the accumulated data to the destination node after the amount of data accumulated on a fixed node exceeds the threshold. From this result, the delay consists mostly of accumulated time. The accumulated time can be suppressed by controlling the threshold at each fixed node. However, since a small threshold causes highly frequent data transmissions, the efficiency of sensing may become low.

Thus, the threshold should be appropriately set according to the system parameters and requirements When ***υ****_m_* is very small, the accumulated time becomes much larger because, mobile nodes spend much time to transfer the acquired data to the fixed node. This makes the rate of data accumulation low. Moreover, the delivery time decreases when ***υ****_m_* increases, which is an obvious result.

#### 4.2.4. Control traffic

**Figure 15.**
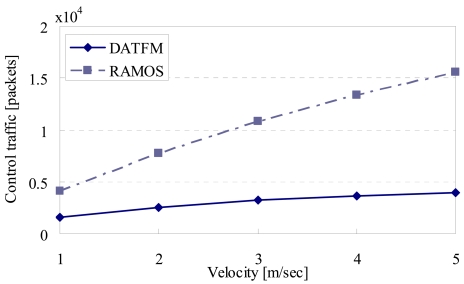
Velocity and control traffic.

Figure 15 shows that the control traffic in DATFM is smaller than that in RAMOS. This is due to the difference of frequency of data transmissions between DATFM and RAMOS. Specifically, the control traffic in RAMOS occurs every time nodes in category3 connect to those in category2 which hold data. On the other hand, the control traffic in DATFM occurs only when a fixed node starts constructing a communication route.

### 4.3. Effects of the communication range

**Figure 16.**
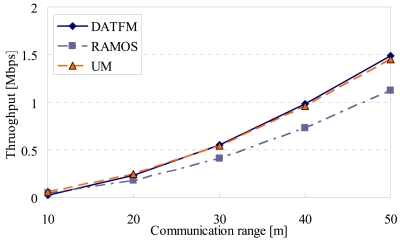
Communication range and throughput.

**Figure 17.**
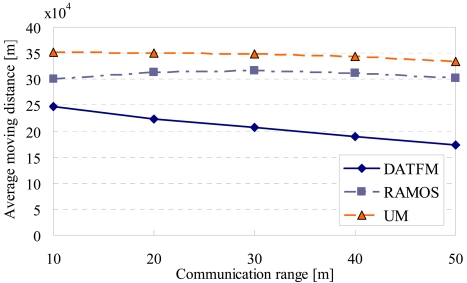
Communication range and average moving distance.

**Figure 18.**
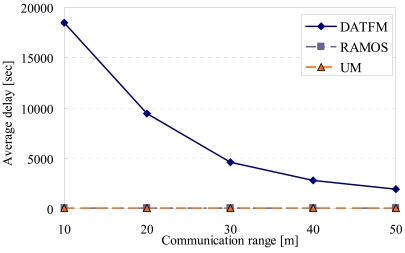
Communication range and average delay.

Figures 16, 17, 18 and 20 show the simulation results changing parameter ***R****_com_*. The horizontal axis on all graphs indicates the communication range of sensor nodes (***R****_com_*). The vertical axis indicates throughput in Figure 16, average moving distance in Figure 17, average delay in Figure 18, and control traffic in Figure 20.

#### 4.3.1. Throughput

Figure 16 shows that the throughputs in all methods get larger as ***R****_com_* increases. This is obvious because the large communication range makes the connectivity high. The difference in throughput between DATFM and RAMOS becomes larger as ***R****_com_* increases. In DATFM, the required number of mobile nodes to construct a communication route decreases. This makes it easy for mobile nodes to return to a sensing operation. On the other hand, the throughput in UM is nearly identical to that in DATFM. This may be due to the following two reasons: a) the number of mobile nodes in DATFM (40) is smaller than that in UM (40+10), and b) the mobile nodes in DATFM can perform sensing operations more frequently than those in UM since they do not move to the sink node to transfer data every time. These are the advantages of UM and DATFM, respectively. In the parameter setting in this subsection, these advantages are balanced out.

#### 4.3.2. Average moving distance

Figure 17 shows that the moving distances in RAMOS and UM are larger than that in DATFM. This is due to the same reasons as that in Figures 12. In addition, as ***R****_com_* gets larger, the average moving distance in DATFM decreases. This is because the required moving distances between the source and destination nodes in DATFM decrease. Moreover, the increase of the communication range makes the moving distance required for the train transmission smaller.

The moving distance in RAMOS increases as the communication range gets longer until a certain value (about 36[sec]). This is because nodes in category3 have more opportunities to connect to nodes in other categories due to the increase of the communication range. On the other hand, as the communication range gets much larger, the moving distance decreases. This is because the moving distance of nodes in category3 required to transfers the data decreases.

The moving distance in UM decreases as the communication range gets larger. This is obvious because the UM nodes can transfer the acquired data with the shorter distance when the communication range is large.

#### 4.3.3. Average delay

Figure 18 shows that the delay in DATFM is always larger than those in RAMOS and UM. This is due to the same reasons as that in Figures 13.

**Figure 19.**
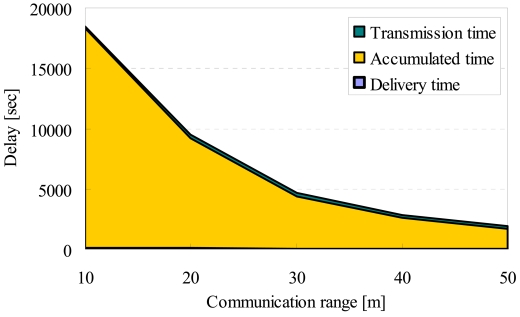
Communication range and details of delay in DATFM.

Figure 19 shows the details of delay in DATFM. This result shows that the accumulated time much increases as ***R****_com_* gets smaller. This is because of the increase of the required number of mobile nodes to achieve a data transmission between fixed nodes. When the required number of nodes increases, many mobile nodes tend to keep transferring data between fixed nodes. Therefore, the number of nodes which perform a sensing operation becomes smaller, and thus, the rate of data accumulation on a fixed node becomes low.

#### 4.3.4. Control traffic

**Figure 20.**
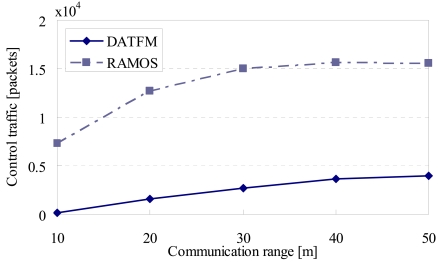
Communication range and control traffic.

Figure 20 shows that the control traffic in DATFM is smaller than that in RAMOS. This is due to the same reasons as that in Figures 15. In addition, the control traffic in DATFM increases as ***R****_com_* get larger. This is because, the larger the communication range is, the faster the fixed nodes accumulate data. Thus, the frequency of data transmissions becomes high. This can be seen from the small accumulated time in Figure 19.

On the other hand, the control traffic in RAMOS increases as ***R****_com_* gets larger. This is because sensor nodes in category3 have more opportunities to connect to nodes in other categories.

**Figure 21.**
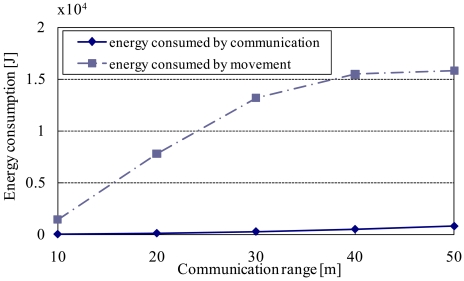
Communication range and the energy consumption in DATFM.

Here, the communication range greatly affects the energy consumed by communication. Thus, in addition to the above criteria, we measure the energy consumed in data transmission processes in DATFM when changing the nodes' communication range. The result is shown in Figure 21.

#### 4.3.5. Energy consumption

As described in the beginning of this subsection, the communication range affects the energy consumed by communication. In addition, the communication range also affects the energy consumed by movement since the moving distance changes according to the communication range as shown in Figure 17. To analyze the effects of communication range to the total energy consumption, we measure the energies consumed by each of communication and movement in data transmission process in DATFM.

In this experiment, we assume that the energy consumed by movement is 1 [J/m], and those consumed by data sending ***P****_s_*(***k, R****_com_*) and receiving ***P****_r_*(***k***) are represented by the following equations [17]:

(13) $${\text{P}}_{\text{s}}(\text{k},{\text{R}}_{\text{com}})=(\text{k}\cdot 50)+(0.1\cdot \text{k}\cdot {\text{R}}_{\text{com}}2).$$

(14) $${\text{P}}_{r}(\text{k})=\text{k}\cdot 50.$$

Here, ***k*** [bit] is the size of the transmitted/received data.

Figure 21 shows the simulation result when changing parameter ***R****_com_*. From this figure, energy consumed by communication increases as the communication range gets larger. In addition, energy consumed by movement also increases as the communication range gets larger. This is because the decrease of the number of mobile nodes to construct the communication route due to the increase of the communication range makes the number of mobile nodes in *SM* larger. In such a case, more data will be accumulated to fixed nodes in a short time, and the frequency of data transmission processes (including train transmission) increases.

Here, this figure also shows that movement of nodes consumes much more energy than communication. This indicates that movement is dominant in power consumption in our assumed environment. As shown in Figure 17, since the average moving distance in DATFM is always smaller than conventional methods, we can confirm that DATFM reduces the total power consumption.

### 4.4. Summary

From the above results, we can see that DATFM can transfer data with smaller moving distance compared with UM and RAMOS. This means that mobile nodes in UM and RAMOS must move for longer distance in order to transfer the acquired data to the sink node. In mobile sensor networks, the energy consumed by movement is much larger than those by communication and computation [17, 18]. Therefore, we can see that DATFM achieves both high throughput and energy-efficiency compared with UM and RAMOS.

Moreover, from the results in Subsection 4.3, we can see that the performance of DATFM is affected by the density of nodes in the network. Here, the density also changes when the number of nodes changes. Although the results are not shown in this paper, we have confirmed by some experiments that the number of nodes has the same effect as the communication range.

## 5. Conclusions

In this paper, we have proposed an effective mobile sensor control method named DATFM for sparse sensor networks. DATFM uses two types of sensor nodes, fixed node and mobile node, and accumulates the acquired data on a fixed node before transferring them to the sink node. In addition, DATFM transmits the accumulated data efficiently by constructing a communication route of mobile nodes between fixed nodes. We have also conducted simulation experiments to evaluate the performance of DATFM. From the results of the experiments, DATFM can acquires and transfers data with smaller moving distance. These results show that DATFM improves the efficiency of sensing and data transmission compared with the conventional methods.

Since each fixed node in DATFM accumulates data generated in its vicinity, the performance of sensing depends on the deployment of fixed nodes. In addition, since the distances between fixed nodes affect the number of required mobile nodes to construct a communication route, the performance of data transfer also depends on the deployment of fixed nodes. Thus, as part of our future work, we plan to discuss the effects of the locations of fixed nodes in order to improve the performance of data transfer.

On the other hand, since mobile nodes cannot sense their target locations when they are transferring the data accumulated by a fixed node, the amount of acquired data tends to decrease in a particular region where data transmissions frequently occur. Such a skew of data acquisition is not desirable in some applications such as planetary explosion and detecting dangerous regions. Thus, we plan to investigate the effects of data transfers in order to achieve the uniform sensing in the whole area while keeping high effectiveness of the data acquisition and transmission.

In DATFM, data transmission is treated as more important than sensing. For example, a mobile node in *SM* which receives a *RCReq* changes its mode into *TM* even when it is performing a sensing operation at its sensing point. However, in some environments, the performance may improve by treating sensing as more important than data transmission. For example, when communication range is large, the required number of mobile nodes for a data transmission process decreases. In such a case, total throughput may be improved when some mobile nodes in *SM* continue sensing operations even when they receive *RCReq*s. Thus, we plan to analyze the effects of the system parameters to the performance, and extend our method to change the behaviors of nodes according to the environment.

Furthermore, in order to adapt to a harsh environment, we also plan to extend our method to handle node failures. Moreover, we plan to examine the effects of difference in memory spaces between mobile and fixed nodes. Finally, we plan to implement our proposed method on real sensor nodes and verify its effectiveness on a practical platform.

## Figures and Tables

**Table 1.** Information of mobile nodes held by a fixed node.

**Node ID**	**Connected time**	**Next fixed node**	**Destination**

a	1,232	*E*	(832,324)
c	1,266	*F*	(542,255)
f	1,335	*E*	(832,324)
i	1,552	*C*	(754,743)
o	1,632	*C*	(754,743)

**Table 2.** Parameters in the experiments.

**Parameter**	**Value (Default)**

***R****_com_*	**10 ∼ 50** (50)
***υ****_m_*	**1.0 ∼ 5.0** (5.0)
